# Possible relationship between fibrosis of IgG4-related thymitis and the profibrotic cytokines, transforming growth factor beta 1, interleukin 1 beta and interferon gamma: a case report

**DOI:** 10.1186/s13000-018-0684-1

**Published:** 2018-01-17

**Authors:** Atsuko Masunaga, Fumihiro Ishibashi, Eitetsu Koh, Takashi Oide, Yasuo Sekine, Kenzo Hiroshima

**Affiliations:** 1 Department of Pathology, Tokyo Women’s Medical University Yachiyo Medical Center, 477-96 Owada-Shinden, Yachiyo, Chiba, 276-8524 Japan; 2 Respiratory Disease Center, Showa University Northern Yokohama Hospital, Yokohama, Kanagawa Japan; 3 Division of Thoracic Surgery, Department of Surgery, Tokyo Women’s Medical University Yachiyo Medical Center, Yachiyo, Chiba, Japan

**Keywords:** IgG4-related disease, Sclerosing mediastinitis, Thymitis, Transforming growth factor beta 1, Interleukin 1 beta, Interferon gamma, Regulatory T cells, Immunohistochemistry, Reverse transcription quantitative polymerase chain reaction

## Abstract

**Background:**

IgG4-related disease often forms a mass and the affected lesion is clinically removed because the mass cannot be differentiated from a neoplasm. Affected lesions commonly occur in the pancreas, hepatobiliary tract, kidney, and retroperitoneum. However, the lesion rarely occurs in the thymus. A histological worldwide consensus of IgG4-related disease proposed that pathological diagnosis of IgG4-related disease should meet more than two of three major features: 1) dense lymphoplasmacytic infiltration with greater than 40% IgG4+/IgG+ plasma cells, 2) storiform fibrosis; and 3) obliterative phlebitis. Currently, fibrosis of IgG4-related disease is thought to be induced by profibrotic cytokines such as transforming growth factor beta 1 (TGFB1), interleukin 1 beta (IL1B) and interferon gamma (IFNG), which are secreted by regulatory T cells (Tregs) and CD4-positive cytotoxic T cells. However, it is unclear whether profibrotic cytokines are associated with the fibrosis seen in IgG4-related thymitis. Here we examined whether cytokines in the mass were increased compared with those in the surrounding thymus, and whether Tregs were present in the mass, using reverse transcription absolute quantitative polymerase chain reaction (RT-ab-qPCR) and immunohistochemistry.

**Case presentation:**

A 70-year-old Japanese man contracted IgG4-letated thymitis. Histological and immunohistochemical analyses demonstrated his mass had massive fibrosis with a focally storiform pattern and lymphoplasmacytic infiltration with 40% IgG4+/IgG+ plasma cells, but not obliterative phlebitis. The mass was surrounded by atrophic thymus. We diagnosed the mass as IgG4-related thymitis. Immunohistochemically, Tregs were scattered throughout the mass. RT-ab-qPCR showed that messenger RNA expressions of TGFB1, IL1B and IFNG in the mass were 270-, 158- and 5.5- fold higher than in the surrounding thymus. His serum IgG4 level after surgery was within the normal range (83.4 mg/dl soon after surgery, 89.3 mg/dl 2 weeks after surgery).

**Conclusions:**

Our results suggested the profibrotic cytokines TGFB1, IL1B and IFNG induce fibrosis and that Tregs might produce some of these cytokines in IgG4-related thymitis as well as in the other affected lesions of IgG4-related disease.

## Background

Sclerosing mediastinitis is a rare disorder and results from many etiologies, e.g. infection, sarcoidosis, radiation and autoimmune conditions [1]. A few cases of sclerosing mediastinitis were reported to be IgG4-related diseases [2–4]. IgG4-related disease shows storiform fibrosis with IgG4-positive plasma cell-rich lymphoplasmacytic infiltrates by histological and immunohistochemical analysis, but high IgG4 levels are not always present in patient sera [4]. IgG4-related mass-forming diseases occur at any site in the body such as the pancreas, biliary duct, salivary gland, kidney, thyroid, aorta, retroperitoneum and mediastinum [4–6]. The masses are clinically recognized and removed as neoplasms [6]. In 2011, IgG4-related disease experts developed IgG4-related disease diagnostic criteria, in which pathological diagnosis of IgG4-related disease requires more than two of three major features: 1) dense lymphoplasmacytic infiltration with greater than 40% IgG4+/IgG+ plasma cells; 2) at least focally arranged storiform fibrosis; and 3) obliterative phlebitis [4]. Recently, Umehara et al. proposed comprehensive diagnostic criteria of IgG4-related disease. According to their new criteria, diagnosis requires the following three condition: 1) organ involvement; 2) > 135 mg/dl IgG in patient serum; and 3) > 40% IgG4+/IgG+ plasma cells in the affected organ [7]. They also reported that serum IgG4 levels are not specific, and that histopathological features are a more specific and important hallmarks of IgG4-related disease [7].

Recent studies have examined the mechanisms through which fibrosis occurs in IgG4-related diseases of the salivary gland, hepato-bilio-pancreatic systems and kidney [7–10]. They speculated that regulatory T cells (Tregs) secreting transforming growth factor beta 1 (TGFB1) in IgG4-related lesions cause mass-forming fibrosis. FoxP3 is a specific marker of Tregs, and two subsets of Tregs secrete TGFB1 and one subset secretes interferon-gamma (IFNG) [11]. Recently, CD4-positive cytotoxic T cells (CTLs), which do not have a specific marker [12], were expanded in the affected lesion of IgG4-related disease of the pancreas, salivary glands, orbit, lymph node, retroperitoneum, kidney and lung [13–16] and secreted the profibrotic cytokines TGFB, interleukin 1 beta (IL1B) and IFNG [12]. However, it is unclear which mechanisms are involved in the fibrosis of IgG4-related sclerosing mediastinitis.

We experienced a case histopathologically consistent with IgG4-related thymitis, which occurred in a thymus of a 70-year-old Japanese man. We assumed that fibrosis of the IgG4-related lesion might be caused by the same mechanism involved in IgG4-related disease of previously examined organs. We evaluated whether Tregs were present and whether TGB1, IL1B and IFNG were increased in the mass of our case using immunohistochemical analysis of FoxP3, and reverse transcription- absolute quantitative polymerase chain reaction (RT- ab-qPCR) for TGB1, IL1B and IFNG messenger RNAs (mRNAs) from paraffin-embedded specimens of our case.

## Case presentation

**Fig. 1:**
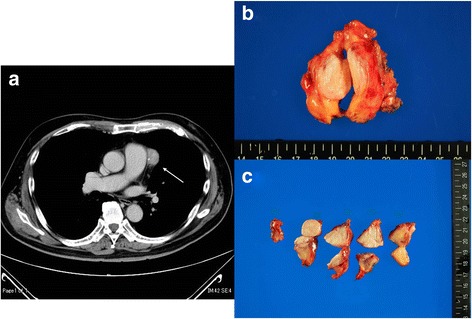
Computed tomography scanning and the removed specimen. **a** Preoperative computed tomography revealed a mass adjacent to the left side of the left pulmonary artery (arrow). However, there was a thin line between the mass and the pulmonary artery. **b** The resected specimen with one section showed a solitary mass surrounded by fat tissue. **c** The cut section: the mass was lobular but with no obvious fibrous septa

A 70-year-old Japanese man was admitted to our hospital because of mediastinal mass found during a routine health examination. He had also suffered from diabetes mellitus, but the disease was fully controlled through medication. Computed tomography (CT) scanning revealed a solid mass measuring 3 cm in the maximum diameter, located adjacent to the left side of the left pulmonary artery. Small calcification was found in the mass (Fig. 1a). Preoperative examination revealed no extra-mediastinal manifestation. We clinically suspected the mass might be a thymoma, and decided to remove the mass. Preoperative examination of his blood and serum detected no abnormal data, although IgG4 was not tested. He did not receive blood transfusion for operation and his loss of blood during operation was 28 ml. His serum was collected soon after surgery and contained 83.4 mg/dl of IgG4 (normal range: 4.8–105 mg/dl) and 1343 mg/dl of IgG (normal range: 800–1700 mg/dl), and IgG fractionation was within the normal range.

The resected specimen was macroscopically a solid and fibrous mass surrounded by fatty tissue (Fig. 1b). Sections showed a whitish and fibrous mass located in the center of the specimen. The mass seemed to be lobular but with no obvious fibrous septa (Fig. 1c).

**Fig. 2:**
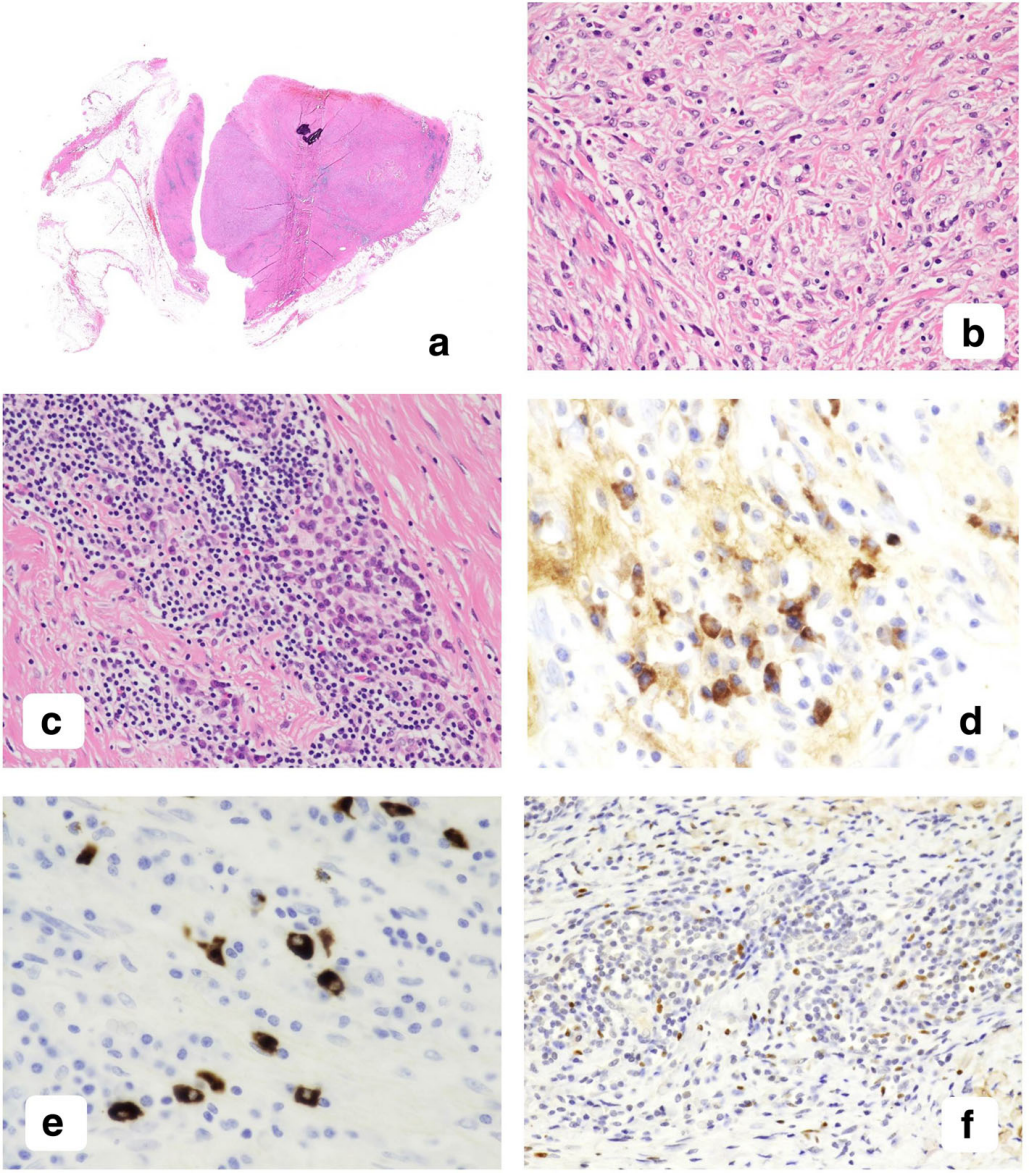
Pathological images with hematoxylin and eosin staining and immunohistochemical staining. **a** A fibrous tumor with inflammatory cells and two foci of calcification. (hematoxylin and eosin (HE), loupe). **b** High magnification view of the tumor shows storiform pattern by fibroblast-like cells. (HE, × 200 magnification). **c** Inflammatory cell aggregates were composed of small lymphocytes and plasma cells (HE, × 200 magnification). **d** IgG-positive plasma cells were located in the inflammatory cell aggregates. (× 400 magnification). **e** Forty percent of IgG-positive plasma cells expressed IgG4. (× 400 magnification). **f** Tregs, which express FoxP3, were scattered throughout the mass. (× 200 magnification)

Hematoxylin and eosin staining analyzed with a loupe showed a pinkish mass surrounded by mature fat tissue (Fig. 2a). Calcification was observed in the mass. Microscopically, the solid mass was composed of dense collagen, fibroblast-like spindle cells, some of which had a large nucleus, and inflammatory cells. No capsulation was seen. Fibroblast-like cells formed a storiform pattern (Fig. 2b), and hyalinized stroma was focal. The inflammatory cells included many small lymphocytes and plasma cells but few eosinophils. The lymphocytes and plasma cells had tendency to aggregate (Fig. 2c), while eosinophils were scattered throughout the mass. There were some plasmablasts, which had immature nuclei with some nucleoli and a narrow perinuclear halo, in the lymphoplasmacytic aggregates. There was no lymphoplasmacytic obliterative phlebitis in the mass by the Elastica van Gieson staining analysis. The histological findings indicated the differential diagnosis of IgG4-related disease, sclerosing thymoma, a solitary fibrous tumor and an inflammatory myofibroblastic tumor [17–19].

Immunohistochemical analyses were performed on paraffin-embedded materials using Bond-Max autostainer (Leica Biosystems Melbourne Pty, Ltd., Melbourne, Victoria, Australia). The mass was negative for keratin, signal transducer and activator of transcription 6 (STAT6), desmin, smooth muscle actin and anaplastic lymphoma kinase (ALK), and there were no terminal deoxynucleotidyl transferase (TdT)-positive immature lymphocytes in the mass. We did not consider the mass as a thymic epithelial tumor such as a thymoma, a solitary fibrous tumor and an inflammatory myofibroblastic tumor accordingly. Almost two thirds of plasma cells were positive for IgG and the mean percentage of IgG-positive plasma cells counted in three × 400 high power fields containing the highest number of IgG4 plasma cells was 40% (Fig. 2d and e). The surrounding fatty tissue contained aggregates of small lymphocytes that were positive for CD3, CD1a and TdT, and that were trapped in keratin-positive meshworks. Consistently, we observed that the surrounding tissue around the mass was an atrophic thymus. According to the histological criteria of IgG4-related disease reported by Deshpande et al. [4], we histopathologically diagnosed the mass as IgG4-related thymitis because the lesion was located in the thymus. Next, immunohistochemically analysis of FoxP3 demonstrated Tregs were scattered throughout the mass (Fig. 2f). We purchased antibodies or antiserum against keratin (clone AE1 and AE3), desmin, smooth muscle actin, CD3, CD1a, TdT and ALK from Leica Biosystems Newcastle Ltd. (Newcastle upon Tyne, UK), against IgG4 from The Binding Site Ltd. (Birmingham, UK), against STAT6 from Santa Cruz Biotechnology, Inc. (Dallas, TX, USA) and against FoxP3 from Cell Signaling Technology (Danvers, MA, USA).

We used RT- ab-qPCR to compare the relative copy numbers of TGFB1, IL1B and IFNG mRNAs to TATA box-binding protein (TBP) mRNA of the mass with those of the surrounding thymus. We isolated total RNA from paraffin-embedded specimens of the mass and the surrounding thymus, using a miRNeasy FFPE Microkit (Qiagen GmbH, Hilden, Germany). Then, the isolated RNAs were reverse-transcribed using PrimeScript II first strand cDNA Synthesis Kit (Takara Bio Inc., Kusatsu, Shiga, Japan). RT-ab-qPCR primer pair for TBP mRNA was the same as previously reported [20], and primer pairs for TGFB1, IL1B and IFNG mRNAs for RT-ab-qPCR were generated according to National Center for Biotechnology Information (NCBI) reference sequences NM_000660.6, NM_000576.2, and NM_000619.2, respectively. The primer pairs are listed in Table 1.We carried out quantitative PCR using the SYBR Green intercalation method with Illumina Eco (San Diego, CA, USA). We confirmed the specificity of every primer pair, seeing one sharp peak on a dissociation curve. The relative expression level of TGFB1 mRNA to TBP mRNA of the mass was 16.2, whereas that of the surrounding thymus was 0.06. The relative expression level of IL1B mRNA to TBP mRNA of the mass was 696.3, whereas that of the surrounding thymus was 4.4. The relative expression level of IFNG mRNA to TBP mRNA of the mass was 79.2, whereas that of the surrounding thymus was 14.5. TGB1, IL1B and IFNG mRNA expression levels of the mass was 270-, 158- and 5.5- fold higher than those of the surrounding thymus, respectively.

**Table 1:** Primer pairs used for reverse transcription quantitative polymerase chain reaction

Target			Amplicon size	Annealing temperature
*TBP*	F	5’-TATAATCCCAAGCGGTTTGC-3’	170 bp	63 °C
	R	5’-GCTGGAAAACCCAACTTCTG-3’		
*TGFB1*	F	5’-GTACCTGAACCCGTGTTGCT-3’	181 bp	63 °C
	R	5’-CAACTCCGGTGACATCAAAA-3′		
*IL1B*	F	5’-GAAGTGCTCCTTCCAGGACC-3’	122 bp	64 °C
	R	5’-TGTCCATGGCCACAACAACT-3’		
*IFNG*	F	5’-CTTGGCTTTTCAGCTCTGCA-3’	120 bp	65 °C
	R	5’-TCCGCTACATCTGAATGACCTG-3’		

F forward primer, *IFNG* interferon-gamma, *IL1B* interleukin 1 beta, R reverse primer, *TBP*, TATA box-binding protein, *TGFB1* transforming growth factor beta 1

Two weeks after surgery, the serum IgG4 of the patient was 89.3 mg/dl and the IgG was 1490 mg/dl. Fractionation of IgG was within the normal range. He was doing well 2 months after surgery.

## Discussion and conclusions

We experienced a rare case of IgG4-related thymitis. In a broad sense, this case is included in sclerosing mediastinitis, because the thymus is located in the anterior mediastinum. However, it is uncertain whether, in the reported cases, the IgG4-related sclerosing mediastinitis occurred in the thymus or not. Our case is thought to be a first case in which IgG4-related lesions undoubtedly occurred in the thymus. Although our patient did not present high IgG4 in his serum, we diagnosed the mass as IgG4-related disease according to a worldwide histopathological consensus reported by Deshpande et al. [4]. IgG4-related disease forms a mass with a focally or diffusely storiform fibrosis, and the masses are often removed because they are clinically recognized as neoplasms. Umehara et al. recently proposed comprehensive criteria of IgG4-related disorders [7]. According to their new comprehensive criteria, a definitive diagnosis of IgG4-related disease that occurs outside the pancreas, the bile duct, the kidney, the respiratory systems and the ophthalmic systems, should be made in the patients with three conditions: organ involvement, high serum IgG4 (> 135 mg/dl) and histological features as IgG4 + plasma cells > 10/high power field and IgG4+/IgG+ cells > 40% in the affected lesion. However, they showed reported IgG4-related cases which had occurred in the skin, the retroperitoneum and the prostate did not present high serum IgG4. There are reported a few cases of IgG4-related sclerosing mediastinitis/thymitis. Inoue et al. reported a case of IgG4-related mediastinitis and that their patient, whose IgG4 of the serum was 127 mg/dl, had been successfully treated with prednisolone. It is uncertain whether the recent criteria of IgG4-related disease proposed by Umehara et al. can be applicable for IgG4-related sclerosing mediastinitis/thymitis or not. Moreover, Fox and Fox reviewed that elevated IgG4 levels in the serum was no longer a surrogate marker fot IgG4-related disease [21], and Wallace et al. reported that elevated counts of circulating plasmablasts were a useful biomarker for diagnosis of IgG4-related disease, even in patients with normal serum IgpG4 concentrations [22]. We did not examine the circulating plasmablasts counts before operation. Therefore, we regarded the worldwide histological consensus of IgG4-related disease more important than the recent criteria of IgG4-related disease.

Currently, the profibrotic cytokines TGFB1, IL1B and IFNG secreted by Tregs and CD4-positive CTLs are thought to induce fibrosis in IgG4-related disease in hepato-bilio-pancreatic systems, salivary glands, retroperitoneum, kidney and lung [8–10, 12–15]. However, it is unclear which mechanisms are involved in the formation of the fibrous mass in IgG4-related thymitis. Here we examined whether Tregs and profibrotic cytokines are associated with the fibrosis of IgG4-related thymitis. We did not evaluate CD4-positive CTLs from this examination because a specific marker for CD4-positive CTLs has not been identified [12]. Our immunohistochemical analysis of Tregs, using antibody against FoxP3, a specific marker of Tregs, showed scattered Tregs throughout the mass. The existence of Tregs in the mass suggested they might secrete profibrotic cytokines. RT-ab-qPCR demonstrated the higher expression of TGFB1, IL1B and IFNG mRNAs in the affected mass compared with the surrounding thymus suggesting Tregs secrete profibrotic cytokines. To confirm the increase in profibrotic cytokines in the mass, the amount of profibrotic cytokine *proteins* extracted from fresh or snap-frozen tissues in the mass should be compared to those in the surrounding thymus. However, we did not take fresh material from the thymus although we saved snap-frozen material from the mass. Consequently, we measured the profibrotic cytokine mRNA expressions. Although epigenetic modification occurs post-transcriptionally [23], our results indicated that profibrotic cytokines might be increased in the mass and related to fibrosis as well as fibrosis of IgG4-related disease in organs other than the thymus.

We did not assess CD4-positive CTLs in the mass. However, increased IL1B mRNA in the mass might indicate the presence of CD4-positive CTLs in the mass, because CD4-positive CTLs in previously examined lesions of IgG4-related disease were reported to secrete TGFB1, IL1B and IFNG that induced fibrosis [12–15].

In conclusion, we showed that increased profibrotic cytokines, TGFB1, IL1B and IFNG might induce fibrosis in the affected lesion of IgG4-related thymitis. However, future studies of fibrosis of IgG4-related thymitis should measure cytokine proteins and ensure that Tregs as well as CD4-positive CTLs isolated from the affected lesion can produce the cytokines and induce fibrosis in vivo and/or in vitro.

## Abbreviations

ALK: Anaplastic lymphoma kinase
CD: Cluster of differentiation
CT: Computed tomography
CTLs: Cytotoxic T cells
IFNG: Interferon gamma
IgG: Immunoglobulin G
IL1B: Interleukin 1 beta
mRNA: Messenger ribonucleic acid
NCBI: National Center for Biotechnology Information
RT-ab-qPCR: Reverse transcription absolute quantitative polymerase chain reaction
STAT6: Signal transducer and activator of transcription 6
TBP: TATA box-binding protein
TdT: Terminal deoxynucleotidyl transferase
TGFB1: Transforming growth factor beta 1
Tregs: Regulatory T cells
